# Prenatal Ablation of Nicotinic Receptor alpha7 Cell Lineages Produces Lumbosacral Spina Bifida the Severity of Which is Modified by Choline and Nicotine Exposure

**DOI:** 10.1002/ajmg.a.35372

**Published:** 2012-03-29

**Authors:** Scott W Rogers, Petr Tvrdik, Mario R Capecchi, Lorise C Gahring

**Affiliations:** 1 Salt Lake City VA Geriatric Research, Education and Clinical CenterSalt Lake City, Utah; 2 Department of Neurobiology and Anatomy, University of UtahSalt Lake City, Utah; 3 Department Human Genetics, University of UtahSalt Lake City, Utah; 4 Howard Hughes Medical InstituteSalt Lake City, Utah; 5 Division of Geriatrics, Department of Internal Medicine, University of UtahSalt Lake City, Utah

**Keywords:** nicotinic receptor alpha7, prenatal development, spina bifida, folic acid, choline, nicotine, mouse genetics

## Abstract

Lumbosacral spina bifida is a common debilitating birth defect whose multiple causes are poorly understood. Here, we provide the first genetic delineation of cholinergic nicotinic receptor alpha7 (*Chrna7*) expression and link the ablation of the *Chrna7* cell lineage to this condition in the mouse. Using homologous recombination, an IRES-Cre bi-cistronic cassette was introduced into the 3′ noncoding region of *Chrna7* (*Chrna7:Cre*) for identifying cell lineages expressing this gene. This lineage first appears at embryonic day E9.0 in rhombomeres 3 and 5 of the neural tube and extends to cell subsets in most tissues by E14.5. Ablation of the *Chrna7:Cre* cell lineage in embryos from crosses with conditionally expressed attenuated diphtheria toxin results in precise developmental defects including omphalocele (89%) and open spina bifida (SB; 80%). We hypothesized that like humans, this defect would be modified by environmental compounds not only folic acid or choline but also nicotine. Prenatal chronic oral nicotine administration substantially worsened the defect to often include the rostral neural tube. In contrast, supplementation of the maternal diet with 2% choline decreased SB prevalence to 38% and dramatically reduced the defect severity. Folic acid supplementation only trended towards a reduced SB frequency. The omphalocele was unaffected by these interventions. These studies identify the *Chrna7* cell lineage as participating in posterior neuropore closure and present a novel model of lower SB that can be substantially modified by the prenatal environment. © 2012 Wiley Periodicals, Inc.

## INTRODUCTION

Neural tube closure in higher vertebrates involves several complex developmental sequences that, if disrupted, can lead to open neural tube defects (NTD). In humans these are relatively common disorders broadly grouped as rostral or cranial NTD (exencephaly or anencephaly) or caudal NTD (spina bifida; SB). Definition of the genetics and the specific mechanisms leading to these defects is complicated by the interaction between the predisposing genes and environmental processes such as maternal diet or toxin exposure.

In humans, environmental modulators of NTD include prenatal dietary supplements such as folic acid and choline [Group, 1991; Enaw et al., 2006], and exposure to various toxins including nicotine as in tobacco use [Kallen, 1998; Shaw et al., 2009a; Suarez et al., 2011]. Cigarette smoking remains a significant health risk during pregnancy [Lynagh et al., 2011] resulting in the exposure of the fetus to an extensive variety of agents including mutagens, carcinogens, hypoxia, and nicotine. A growing number of studies collectively indicate that nicotine itself modulates a wide range of cellular and developmental processes whose impact may not be observed until later in life [Gahring and Rogers, 2005; Nekhayeva et al., 2005; Ginzel et al., 2007; Pauly and Slotkin, 2008; Albuquerque et al., 2009]. However, how nicotine acts upon developmental processes is not resolved, and the relationship between prenatal nicotine exposure and subsequent developmental abnormalities can be obscured by the complexity of the constituents in tobacco. Nicotine acts through modulating the function of the nicotinic acetylcholine receptors (nAChR). The nAChRs are ligand-activated excitatory ion channel receptors whose functional and pharmacological properties are determined by the inclusion of different combinations of genetically distinct alpha or non-alpha (usually beta) subunits into a mature pentameric receptor complex [Albuquerque et al., 2009]. For example, nAChRs containing alpha4 and beta2 subunits bind nicotine with high affinity and participate in the processes leading to addiction. Another receptor is the alpha-bungarotoxin binding nicotinic receptor alpha7 (α7) that is expressed in both neuronal and non-neuronal tissues throughout the body. Its function has been implicated in diverse processes including neurotransmission, gene expression, cell proliferation and survival, cell differentiation, and inflammation [Lauder, 1993; Heeschen et al., 2001; Gahring and Rogers, 2005; Serobyan et al., 2007; Resende et al., 2008; Wessler and Kirkpatrick, 2008]. Thus, one challenge is to distinguish among the potential nAChR targets of nicotine in terms of how nicotine contributes to the origin and severity of dysfunction in these processes during prenatal development.

We approached this problem through methods of homologous recombination to identify and subsequently modify specific nAChRs to define their respective cell lineages and define the phenotypes that arise from their dysfunction. Initially, we have focused on the α7 receptor because it is expressed in multiple tissues throughout the body and its high permeability to calcium ions is sufficient to activate multiple calcium-mediated processes including second messenger systems (e.g., CREB and NfκB signaling), certain proteases and modulate certain voltage-gated channel activity [Albuquerque et al., 2009]. Also, α7 responds to multiple ligands comprising full agonists that in addition to acetylcholine and nicotine include choline from both endogenous and dietary sources [Zeisel, 2006, 2011; Albuquerque et al., 2009; Abreu-Villaca et al., 2011]. To identify the cell lineages that express α7 and examine how their manipulation contributes to developmental processes we applied methods of homologous recombination in mouse embryonic stem cells to introduce an *IRES-Cre* bi-cistronic gene cassette at the 3′ end of the mouse α7 gene, *Chrna7 (Chrna7:Cre)*. In this mouse, cell lineages expressing α7 can be identified and manipulated in crosses with females with genes of interest whose activation or inactivation is controlled by flanking LoxP (floxed) sequences. We report these newly designed mice and the unanticipated finding that genetic ablation of the α7 cell lineage in embryos of the *Chrna7:Cre* × *ROSA26-loxP(DTA)* produced several defects, one of which is lumbosacral spina bifida (SB). Also, present is an abdominal wall defect of omphalocele (extruded liver and intestines omphalocele covered with the peritoneum and amnion). We noted a dramatic increase in SB prevalence and severity in embryos exposed to prenatal nicotine. In contrast, prenatal supplementation with choline decreased occurrence and severity of the SB defect. Folic acid, another dietary anti-NTD agent, had no impact on the frequency of SB, but it did decrease the severity of the defect. The genetic tools generated here provide a tractable model for neurodevelopment defects related to cell lineages expressing *Chrna7* and for the first time indicates these contribute to several developmental processes including caudal neural tube closure. Further, as in humans with SB, there is variability in the incidence and severity that is subject to modification by the prenatal environment and maternal diet.

## MATERIALS AND METHODS

### Animals

The Cre-dependent beta-galactosidase reporter (*ROSA26*-*lacZ*, *R26R*) and the conditional diphtheria toxin (DTA) mouse lines were described previously [Soriano, 1999; Wu et al., 2006]. All animal use was in accordance with the Guide for the Care and use of Laboratory Animals of the National Institutes of Health. Animal protocols were approved in advance by the Institutional Animal Care and Use Committee (IACUC) at the University of Utah. Animals are housed according to NIH and IACUC guidelines.

### Generation of α7*-IRES-Cre* Mice

**FIG. 1:**
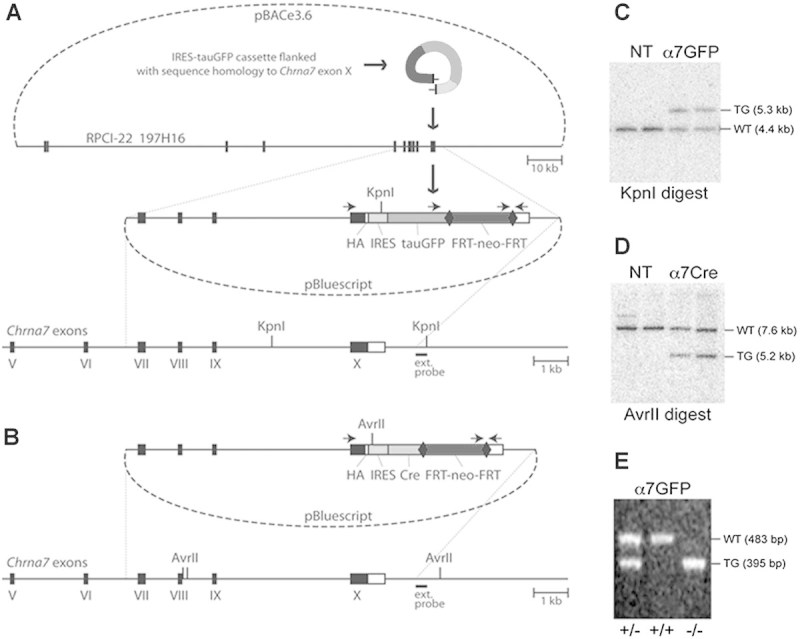
Construction of the targeting vectors and generation of the corresponding mouse lines. **A**: A diagram illustrating the production of *Chrna7*-IRES-tauGFP. In the first step, the IRES-tauGFP-FRT-neo-FRT cassette 1, including the HA epitope, was inserted in the 3′ untranslated region (UTR) of *Chrna7* (Methods). The external hybridization probe position, PCR priming sites and *Kpn*I restriction sites are indicated. Numbered black boxes depict protein-coding exons. HA, hemagglutinin epitope; IRES, internal ribosomal entry site; FRT, recognition target sites of the FLP site-specific recombinase; neo, neomycin (kanamycin or G418 resistance); bovine tau polypeptide fusion to enhanced green fluorescent protein (tauGFP). **B**: The *Chrna7*-IRES-Cre targeting vector was created by replacing tauGFP with the IRES-Cre-FRT-neo-FRT cassette 2. The external hybridization probe was as in (A). **C**: Southern blot showing *Kpn*I restriction fragment size upshift from 4.4 kb (WT, wild type) to 5.3 kb (TG, targeted allele) in the *Chrna7*-IRES-tauGFP targeted ES cell DNA. NT, negative ES clones harboring non-homologous recombination. **D**: *Chrna7*-IRES-Cre and *Avr*II restriction analysis producing an anticipated downshift from 7.6 kb (WT) to 5.2 kb. **E**: PCR genotyping of *Chrna7*-IRES-tauGFP mice in which the FRT-neo-FRT selection cassette was removed (Methods). The PCR data demonstrates the absence of wild type allele in normally segregating homozygous (−/−) *Chrna7*-IREStauGFP/IRES-tauGFP or *Chrna7*-IRES-Cre animals.

We used methods of homologous recombination in mouse embryonic stem cells to modify the mouse α7 gene (*Chrna7*) to co-express the Cre-recombinase. Details of the construction of these mice including sequencing primers are available upon request. Briefly, we initially developed a *Chrna7-IRES-tauGFP* mouse using the RED recombining technology 5 and 129S6/SvEvTac BAC DNA isolated from the RPCI-22 BAC Library 6 (Fig. 1A). BAC clones were isolated and one clone, 197H16 BAC, was electroporated into the EL350 *Escherichia coli* strain to add the hemagglutinin (HA) epitope tag (YPYDVPDY) and stop codon extension of the *Chrna7* polypeptide, to the C-terminus of α7 and the *IRES-tauGFP-FRT-neo-FRT* cassette (Fig. 1A) was added adjacent to the STOP codon in the *Chrna7 gene* using annealed oligonucleotides. After removal of the flanking regions to the HA epitope and the reporter/selection cassette, the fragment was purified and electroporated into recombinogenic EL350 cells containing the 197H16 BAC clone. Successfully recombined BAC vectors were selected on plates containing chloramphenicol (for the pBACe3.6 backbone) and kanamycin (for *EM7*-neo). The final targeting vector with suitable homology arms was prepared using two separate regions of homology complementary to the distal ends of the 5′ and 3′ ends of the targeting region of the 197H16 BAC, digested and inserted in the pBluescript II SK+ plasmid vector. This “pull-down” vector was linearized and electroporated into heat-induced EL350 cells harboring the modified 197H16 BAC. The cells were selected on plates containing ampicillin (for the pBluescript backbone) and kanamycin and clones containing the anticipated 9,440 bp genomic DNA fragment harboring the last four exons of *Chrna7* including the previously made alterations identified. The *Chrna7-IRES-Cre* targeting vector was then cloned by replacing *tauGFP* with an *IRES- Cre-FRT-neo-FRT* cassette using standard cloning techniques (Fig. 1B). Each targeting vector was electroporated into R1 embryonic stem cells and selected with G418. Clones were expanded and analyzed by Southern blotting of genomic DNA. Clones with positive inserts were selected based upon showing the anticipated up-shift from 4.4 kb in the wild type to 5.3 kb in the targeted locus (Fig. 1C) and four α7Cre targets (downshift from 7.6 kb in the wild type to 5.2 kb in the targeted DNA; Fig. 1D).

Mice were next generated from the positive ES clones by blastocyst injection and the chimeric founders were mated with FLPe deleter mice 8 in order to remove the neomycin selection marker. The resulting *Chrna7-IRES-tGFP* or *Chrna7-IRES-Cre* line was subsequently backcrossed to the C57BL/6 background and PCR was performed to confirm expression and genotype (Fig. 1E). An automated genotyping protocol for these alleles was developed by Transnetyx (using probes “*Chrna7*-2 WT,” “eGFP,” and “CRE”).

### Choline, Folic Acid, and Nicotine Supplementation

From the initiation of pregnancy dams were fed Harlan Teklad rodent chow (Harlan, Madison, WI) formulated as either standard choline (0.2% w/w, TD.03118) or choline supplemented (2% w/w, TD.03119) [Guseva et al., 2008]. Folic acid (3 mg/kg in saline; Sigma–Aldrich, St. Louis, MO, F7876) was administered i.p. to pregnant females on a daily basis [Shin and Shiota, 1999; Barbera et al., 2002]. Oral nicotine (base) was administered in the drinking water [Rogers et al., 1998; Matta et al., 2007] with minor modifications. Upon plug identification, dams were placed on 50 µg/ml nicotine, 2% saccharin in water, followed by 100 µg/ml nicotine on days 2–3, and 150 µg/ml nicotine on day 4 for the remaining time. For the folic acid or nicotine treatments, the animals were maintained on the standard choline diet.

### Immunohistochemistry

Embryos or adult tissues were perfused transcardially with saline and fixed in freshly prepared EM-grade 2% or 4% paraformaldehyde (EMS #15713) in 5% sucrose and then cryoprotected in PBS containing 15% and then 30% sucrose. Specimens were embedded in 2% gelatin (Sigma–Aldrich G2500) in normal saline, quick-frozen in liquid nitrogen and sectioned at 10–20 µm with the Thermo Scientific HM 550 Cryostat (ThermoFisher Scientific, Waltham, MA). For staining, sections were mounted on Superfrost Plus Gold slides permeabilized and stained with either anti-peripherin (1:100; EMD Millipore Billerica, MA AB1530) and FITC, Alexa Fluor 488 secondary antibodies (Jackson ImmunoResearch, West Grove, PA). Histological staining of paraffin sections with hematoxylin and eosin (H&E) was performed by ARUP Laboratories (Salt Lake City, UT).

### X-Gal Staining and Histology

Embryos dissected at E8-10 and fixed in 2% paraformaldehyde in PBS, permeabilized in 0.02% NP-40 and 0.01% Na deoxycholate, and stained as a whole mount in a PBS solution containing 1 mg/ml X-gal, 25 mM K_3_Fe(CN)_6_, 25 mM K_4_Fe(CN)_6_ · 3H_2_O, 2 mM MgCl_2_, 0.02% NP-40, 0.01% Na deoxycholate and subsequently cleared.

### Fluorescence Microscopy and Imaging

Fluorescence imaging and digital photography was performed either with a Zeiss Axiovert 200 microscope equipped with an X-cite fluorescent source and a MicroFire CCD camera or a Leica MZ12 stereomicroscope. Digital images were processed with Imaris 6.0 (Bitplane), Adobe Photoshop CS2, or Adobe Illustrator CS3.

### Scanning Electron Microscopy

E11.5 mouse embryos were fixed in 2.5% glutaraldehyde in 0.1 M sodium cacodylate buffer at 4°C overnight, alcohol dehydrated and stained with osmium tetroxide. Imaging was performed with S-2460N Hitachi scanning electron microscope at the Electron Microscopy Core Facility at the University of Utah.

### Statistical Analyses

Statistical evaluations used 2 × 2 contingency table analyses for each treatment and condition that were relative to standard choline controls. Two-tailed Fisher's exact tests were calculated using the GraphPad software.

## RESULTS

**FIG. 2:**
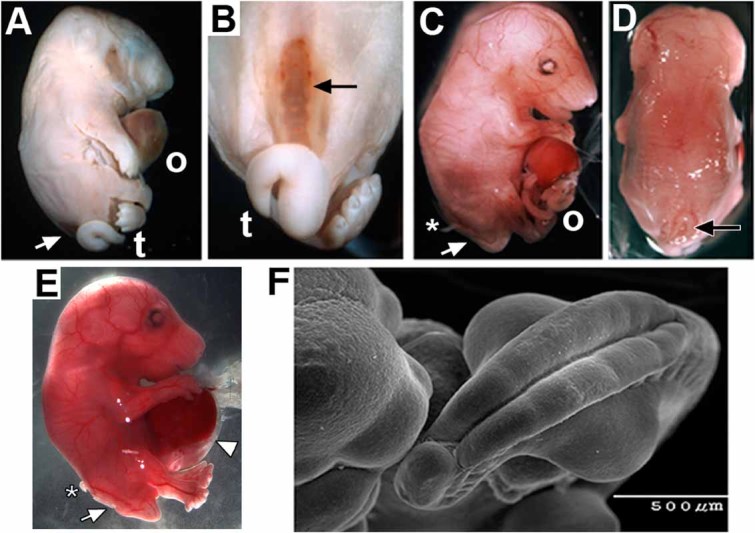
Ablation of the *Chrna7:Cre* cell lineage results in rostral neural tube and ventral body wall defects. **A**: The typical appearance of stillborn mice of the *Chrna7:Cre* × *ROSA26-loxP(DTA)* genotype. Prominent defects include an omphalocele (o) and a ventral neural tube defect (spina bifida (SB) arrow). Also evident is the abnormal tail (t). **B**: Another view showing the open spina bifida and abnormal tail phenotype. **C**: The same defects are present in E16.5 embryos. This includes an omphalocele (o) and spina bifida (arrow). Also present are extruded dorsal root ganglia (asterisk). The surrounding peritoneum was disrupted during preparation of this embryo. **D**: Another view of the same embryo in C illustrating the specificity of the SB defect (arrow). **E**: A side view at E16.5 that shows the extruded liver and intestines covered with the peritoneum and amnion defining an omphalocele (arrow head). Also evident is open SB (arrow) and extruded dorsal root ganglia (asterisk). **F**: A scanning electron micrograph of an E11.5 embryo shows the severe delay of the caudal neuropore closure. Also, note the exaggerated curvature of the caudal axis and reverted tail tip.

We began the examination of how α7 participates in prenatal development by crossing the *Chrna7:Cre* male mice (Methods and Fig. 1) with females harboring the conditional attenuated diphtheria toxin (DTA) construct, *ROSA26-loxP(DTA)*. Pups from these crosses were stillborn (Fig. 2A) with several developmental abnormalities. Overall they had a grossly normal body but they often exhibited a hunched posture, flat head and abnormally proportioned limbs (especially lengthened hind limbs). Most evident was protrusion of abdominal organs (liver and intestines) suggestive of an omphalocele (Fig. 2A). Also prominent was the defect of open spina bifida (SB) in nine of 13 pups from three litters (Fig. 2A–C). This defect was always accompanied by a tail deformity reminiscent of *curly tail* [van Straaten and Copp, 2001; Ting et al., 2003]. We next examined prenatal litters at embryonic day 16.5 (E16.5). The open SB defect and omphalocele were evident (Fig. 2C,D) although neither defect was fully penetrant (Table I; see van Straaten and Copp [2001]; and Harris and Juriloff, [2010]). Also, careful removal of embryos at this stage reveals the abdominal wall defect to include peritoneal membrane enclosing the extruded liver and intestines that is characteristic of omphalocele (Fig. 2E). Additional developmental deficiencies included irregularities in the retinal pigmented epithelium and a severe reduction in dorsal root ganglion sensory neurons (not shown). Craniofacial malformations were rare and generally restricted to the mandible, which was shortened. Hematopoiesis was present as indicated by red blood cells although as many as 30% of the embryos in some litters could be anemic. RNA levels for other nAChR subunits (α2–α6 and β2–β4, respectively) were not altered relative to controls as might be expected if there were significant compensatory mechanisms or if other receptor subtypes contributed to this phenotype (not shown). The location of the embryos in the placental order also failed to correspond with the defect penetrance or severity (not shown).

**TABLE I:** Dietary Effects on the Frequency of Spina Bifida, “Curly Tail” and Omphalocele in *Chrna7:Cre* Ablated Mouse Embryos

Diet	Number embryos (litters)^a^	Avg. litter	Spina bifida n(%)	“Curly Tail” n(%)	Omphalocele n (%)
Standard diet	35 (8)	4.4	28/35 (80)	29/35 (83)	31/35 (89)
Standard diet + oral nicotine	14 (3)	4.7	13/14 (93)	13/14 (93)	12/14 (86)
Standard diet + folic acid	17 (5)	3.4	9/17 (53)	12/17 (71)	14/17 (82)
Standard diet + choline (2%)	26 (6)	4.3	10/26 (38)*	11/26 (42)*	21/26 (81)

a Embryos (E16.5) were harvested, fixed, and scored for anatomical defects. Severely retarded embryos were not included in this sampling because of uncertainties regarding scoring of their phenotype.

* *P* < 0.01 relative to Standard Choline, two-tailed Fisher's exact test.

In earlier embryos, the SB defect was visible as early as E10.5 (not shown) and it was clearly evident in E11.5 embryos as shown in SEM imaging (Fig. 2F). This is also consistent with the closure of the posterior neuropore as the first appreciable developmental defect observed. Caudal ends of the embryos were highly curved and the tips of their tails were bent backwards (not shown), which is somewhat reminiscent of “curly tail” mice where an increased curvature in the sacral and coccygeal region is common [van Straaten and Copp, 2001; Ting et al., 2003].

**FIG. 3:**
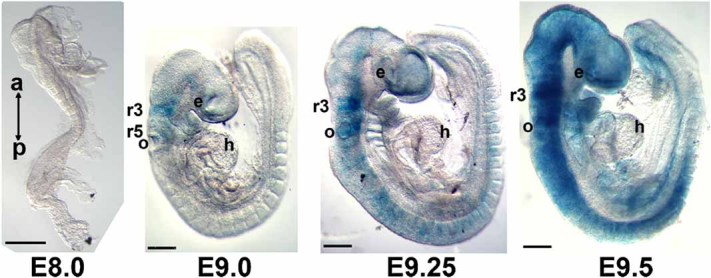
Earliest detection and expansion of the *Chrna7:Cre* cell lineage as defined by *Chrna7:Cre* × *ROSA26-loxP(lacZ)* alleles and X-gal staining. The expression of *Chrna7:Cre* is not detected in E8.0 embryos (the anterior (a) to posterior (p) direction is noted), but X-gal is detected at E9.0 where it is restricted to rhombomeres 3 (r3) and 5 (r5) of the developing hindbrain of the recently “turned” embryos. The otic vesicle (o), eye (e), and heart (h) are identified. This expression extends rapidly to other parts of the embryo from E9.25 through E9.5 until X-gal expression is present in cells within most tissues and organs. Scale bars are 1 mm.

To determine if these defects are consistent with the onset of *Chrna7:Cre* expression, we crossed the *Chrna7:Cre* male with the *ROSA26-lacZ* (*R26R*) reporter female mouse. The earliest sites of *Chrna7:Cre* expression were detected by X-gal staining to be in rhombomeres 3 and 5 of the developing hindbrain of E9.0 embryos (Fig. 3). The *Chrna7:Cre* expression domain extends rapidly to include subpopulations of cells in many organs throughout the body by E9.5 (Fig. 3). Because this gene is not expressed until E9.0, no impact of lineage ablation on developmental processes that occur prior to this stage would be anticipated.

**FIG. 4:**
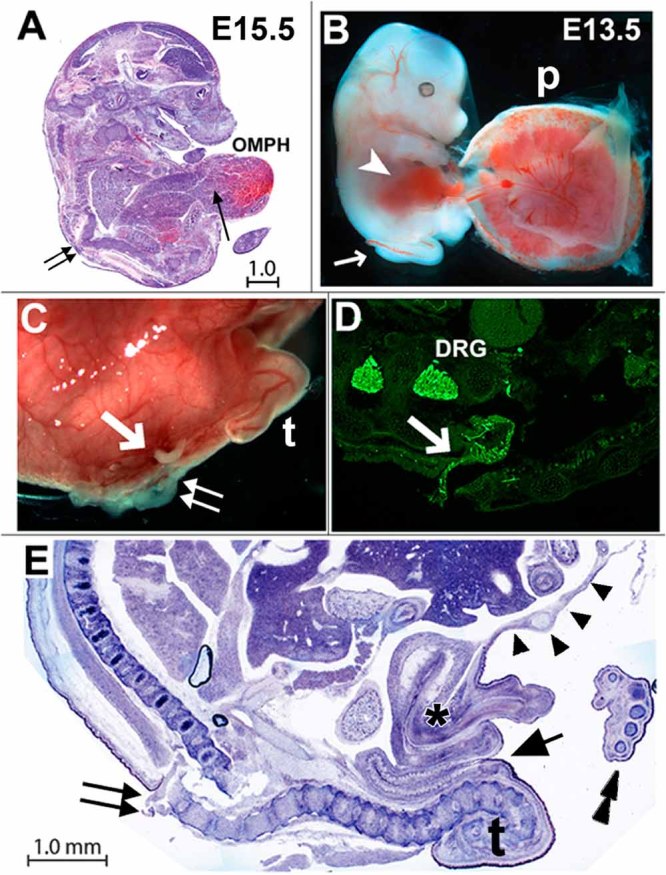
*Chrna7:Cre* ablated embryo defects. **A**: Staining (H&E) of an E15.5 embryo shows the spina bifida defect (double arrows) and omphalocele with extruded liver (OMPH). The proposed overgrowth of the normal liver is seen in the extruded portion of this organ (arrow). **B**: The placenta (p) develops normally (E13.5), although some necrotic conceptuses are regularly observed (not shown). Note that the ventral body wall defect is already visible (arrowhead) as is the spina bifida defect (arrow). **C**: At E16.5, *Chrna7:Cre* ablated embryos frequently manifest myelomeningocele (i.e., extrusion of the meninges and spinal cord) at the site of the SB (double arrows) and nerve roots (arrow). The abnormal tail (t) is also present. **D**: A section through the same embryo stained with antibodies to neural marker peripherin. This highlights the dorsal root ganglia (DRG) and shows the extruded nerve root from one of these (arrow) that is topographically displaced. **E**: Pelvic anatomy of an E16.5 *Chrna7:Cre* ablated fetus. The SB defect and extruded DRG nerve root is identified (double arrows). In these embryos, the urogenital system (asterisk) and anus (arrow) appear unaffected as does the axial autopod skeleton (stacked arrowheads). Notable is the hernia sac and peritoneum membrane surrounding the extruded abdominal organs (arrow heads) that define the omphalocele. This fragile membrane is frequently disrupted during dissection. All scale bars are 1 mm.

Additional methods were used to examine the extent of the *Chrna7:Cre* ablation developmental defects. Gross embryonic morphology is intact and overall resembles the normal body plan as would be expected since *Chrna7:Cre* is not expressed until after E9.0 (Fig. 4A). Disruption of structure in the lower lumbar and sacral region at the eventual site of the SB defect becomes apparent. Also these fetuses appear to have an excessively large liver that is extruded as part of the omphalocele (Fig. 4A). Despite this, the placental organization appears to be grossly normal (Fig. 4B). Other organs are also occasionally seen to be disproportionally large including the pancreas (not shown). Gross defects in tissues at E16.5 include enlarged teeth and salivary glands and absence or disorganization of retinal-pigmented epithelium (Fig. 4A and not shown). The patterning of major brain divisions from forebrain to hindbrain appeared to be overall normal for this stage of development as do basic skeletal and muscle formation (Fig. 4A and not shown). At later developmental stages (E16.5) the splaying of the vertebral processes affects outgrowth of the spinal nerves which is particularly evident when dorsal roots become misdirected and extruded (Fig. 4C,D). Given these defects, we considered the possibility that our model resembled the human condition of omphalocele, bladder exstrophy, imperforate anus, spine defect (OEIS) complex [Carey et al., 1978; Kallen et al., 2000]. However, this seems unlikely since major features of the OEIS complex are absent such as a largely unaffected urogenital system, and the imperforate anus is absent (Fig. 4E). Thus, this mouse presents a unique subset of midline defects [Khoury et al., 1989].

**FIG. 5:**
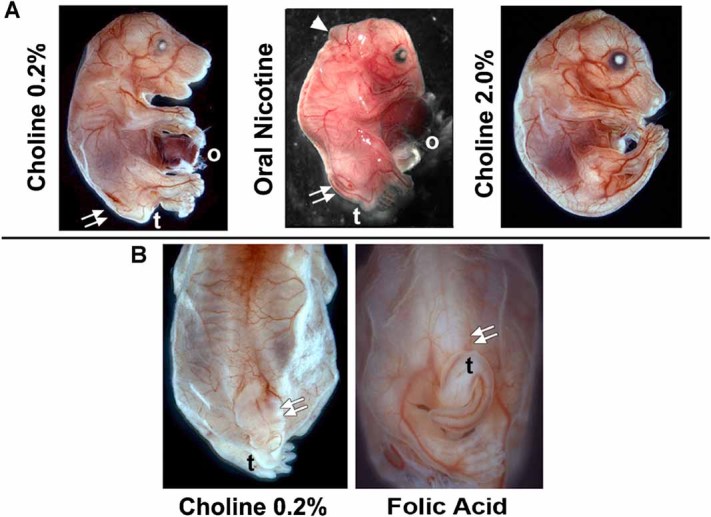
Maternal diet supplemented with oral nicotine, 2% choline or folic acid modifies the severity of spina bifida (SB) in embryos from the *Chrna7:Cre* × *ROSA26-loxP(DTA)* cross. **A**: Typical E16.5 embryo receiving the normal dietary chow containing 0.2% choline. As before, the SB defect (double arrows), tail (t) and omphalocele (o) are identified. For embryos exposed to oral nicotine (Methods) from the time of conception, in addition to the expected defects there were also abnormalities of the rostral tube closure (arrow head) that in some cases was sufficiently severe to include exencephaly (not shown). Also note the reduced torso size typical of these fetuses. Embryos receiving 2% choline supplementation exhibited less severe developmental abnormalities. In this case the omphalocele was also absent, although this was not the case for the majority of these embryos (Table I) despite the reduced prevalence of SB. **B**: Folic acid supplementation trended towards moderation of the SB defect compared with the normal 0.2% choline chow (double arrows). However, the occurrence of the tail defect (t) and overall SB frequency were not significantly rescued. Folic acid supplementation also did not affect the incidence of omphalocele (not shown, see Table I).

As noted in the Introduction section, neural tube closure involves complex interactions between genetic and environmental factors. The partial penetrance and variable severity of SB in the *Chrna7:Cre* lineage ablation mouse, even among litter mates, offers the opportunity to examine if agents that are implicated in moderating NTD severity, including agents that modulate α7 function, affect the prevalence or severity of this defect (Fig. 5). Periconceptional folic acid supplementation produces up to 70% reduction of human neural tube defects [Group, 1991] and this extends to mice [Shin and Shiota, 1999; Barbera et al., 2002; Harris, 2009]. We tested whether daily folic acid administration improved the posterior neuropore closure defect. In five litters examined at E16.5, folic acid supplementation (Methods) trended towards a reduced open SB frequency (Table I) that approached statistical significance (*P* = 0.056), but the tail morphology remained abnormal (Fig. 5B). No effect by folic acid on the omphalocele defect was observed and litter size as measured at E16.5 did not differ among this or other treatment groups and the controls (Table I).

In addition to folic acid, other dietary and environmental agents affect SB in humans and other mouse NTDs [Enaw et al., 2006; Zeisel, 2006; Guseva et al., 2008; Shaw et al., 2009b]. This includes choline (also a one-carbon metabolic donor, [Zeisel, 2009]) or nicotine, both ligands of α7 and both indicated to impact upon the occurrence and severity of certain NTDs [Kallen, 1998; Suarez et al., 2011]. To begin we examined the severity of SB when choline in the maternal prenatal chow was supplemented from 0.2% to 2% (w/w). In all litters examined the frequency of SB was significantly decreased (Fig. 5A; Table I), there was a substantial restoration of tail bud development and the caudal body axis size appeared improved (Fig. 5A; Table I). Thus, the choline-supplemented diet proved dramatically more effective than folic acid in moderating the SB defect. In contrast, prenatal oral nicotine administration (Methods) corresponded to an increase of SB prevalence and severity when examined at E16.5 (Fig. 5A and Table I). The increased severity of the SB was particularly evident in a subset of embryos where the defect extended to the rostral neural tube suggestive of an exencephaly-like phenotype (Fig. 5A). Also evident was an overall reduction in body size that again affected the lower torso more severely leaving the appearance of an enhanced omphalocele. Thus, in this α7-lineage ablation model of SB we observe choline and nicotine have opposite effects on the phenotype prevalence and severity.

## DISCUSSION

We have used homologous recombination to modify the nicotinic acetylcholine receptor alpha7 (*Chrna7:Cre)* transcript to provide a flexible approach for examining the temporal and spatial expression of this receptor in the developing mouse. The expression of *Chrna7:Cre* is detected first in rhombomeres 3 and 5 of the E9.0 mouse embryo and thereafter it extends to include subsets of cells located in tissues throughout the embryo. A notable developmental defect resulting from the conditional DTA ablation of the *Chrna7:Cre* cell lineage is a precise caudal NTD consistent with open spina bifida (SB). The prevalence and severity of this defect is responsive to dietary supplementation with agents that also affect SB frequency in humans. Also evident is the prominent omphalocele, possibly resulting from disproportionately large organs. Thus processes in this mouse leading to the SB and omphalocele defects are mechanistically separable as demonstrated by the differing response of SB to oral nicotine and choline treatment in the absence of a change in the frequency of omphalocele (Fig. 5 and Table I).

The precise nature of the SB defect following ablation of *Chrna7:Cre* lineage cells is particularly intriguing. Of approximately 250 NTD mouse models it is reported that perhaps less than 5% of these exhibit a caudally restricted NTD indicating the mouse favors the formation of more rostral defects [Harris and Juriloff, 2007, 2010]. This suggests a more complicated model than simple cell ablation leading to the defect since early α7 expression is present in the rostral fold fields (Fig. 3) but the processes of tube closure in this region continues (unless nicotine is present). Several explanations seem possible for this observation. One is that the ablation of the *Chrna7:Cre* expressing cell population leads to distortion in nearby structures of the caudal region (consistent with the displaced DRG) and this produces steric constraints that precludes successful closure at this location. Another possibility is suggested by recent studies demonstrating that other cell types can compensate for certain defects of normal caudal development and a timely posterior neuropore closure [Olaopa et al., 2011]. For example, choline could work independently of α7 to favor local enhancement of cell proliferation and thus initiate repair [Zeisel, 2006, 2011]. In contrast, nicotine is well known to modify processes of cell proliferation and wound healing, possibly through interaction with additional receptor subtypes [Wessler and Kirkpatrick, 2008; Martin et al., 2009]. Also possible is that these agents could modify *Chrna7:Cre* transcription [Reynolds and Hoidal, 2005; Albuquerque et al., 2009; Sakurai et al., 2011]. In this scenario, highly localized down-regulation of transcription by choline and increased receptor transcription by nicotine would impact directly upon threshold levels of Cre sufficient to initiate DTA recombination and cell ablation. Both *Chrna7:Cre* lineage ablation and transcript down-regulation would produce results consistent with PCR results indicating no detectable *Chrna7* RNA (not shown). Thus, this is a topic for more detailed examination towards understanding the mechanism of these environmental impacts on this model of SB.

We think it unlikely that *Chrna7* will prove to be a candidate gene for SB since the extensive use of the *Chrna7:*KO mouse under diverse environmental conditions, to our knowledge, has never been associated with a NTD developmental disorder [Orr-Urtreger et al., 1997; Picciotto et al., 2001; Albuquerque et al., 2009]. Instead, we view the likely contribution of α7 dysfunction in NTD as modulatory to other primary mechanisms whose dysfunction predisposes the individual to a NTD such as SB. In particular, the apparently wide-spread expression of *Chrna7:Cre* in cell subsets throughout tissues of the developing fetus and the variety of potential defects in addition to SB (such as omphalocele, hematopoietic disruption, or reduced trunk size as noted in the Results section) indicates that the function of α7 is likely to exhibit considerable pleiotropy. This would be consistent with findings in the adult that this receptor can participate in modulating neurotransmission [Albuquerque et al., 2009], pro-inflammatory responses via both neuronal and non-neuronal local cellular mechanisms [Gahring and Rogers, 2005] and even promote tumor cell growth in the lung [Thunnissen, 2009; Improgo et al., 2011]. Also important is that the functional role of α7 in these processes is not always appreciated unless the system is examined under stress conditions that exceed the modulatory capacity of this receptor. This is particularly the case in the pro-inflammatory mechanisms in the adult immune system where the impact of α7 is best measured under strong inflammatory challenge [Gahring and Rogers, 2005]. Thus, this may be a similar case where the contribution of α7 to these developmental processes is inferred only when the cells that express *Chrna7:Cre* are ablated and fail to contribute to the developmental process in mass. Otherwise, normal perturbations to α7 function due to environmental compounds might produce outcomes that are far more subtle. Further, the tissues and processes impacted upon could be expected to vary considerably based upon the compound and timing or duration of exposure. While greater temporal and spatial resolution that includes sufficient sensitive or additional pharmacological interventions are required to measure individual cell responses and long-term impact upon the defects observed, this model opens multiple experimental avenues towards understanding the mechanisms of α7 contributions to these birth defects. Also this model could contribute towards an understanding of the contradictory literature concerning the impact of prenatal nicotine exposure and diet on SB frequency in geographically dissimilar populations [Frey and Hauser, 2003; Shaw et al., 2009a; Au et al., 2010; Caudill, 2010].
